# Supplementary figures and images for: Tumour-driven lipid accumulation in oenocytes reflects systemic lipid alterations
Source: PLoS Genet. 2026 May 7;22(5):e1012150. doi: 10.1371/journal.pgen.1012150 (PMC13167029; doi:10.1371/journal.pgen.1012150)

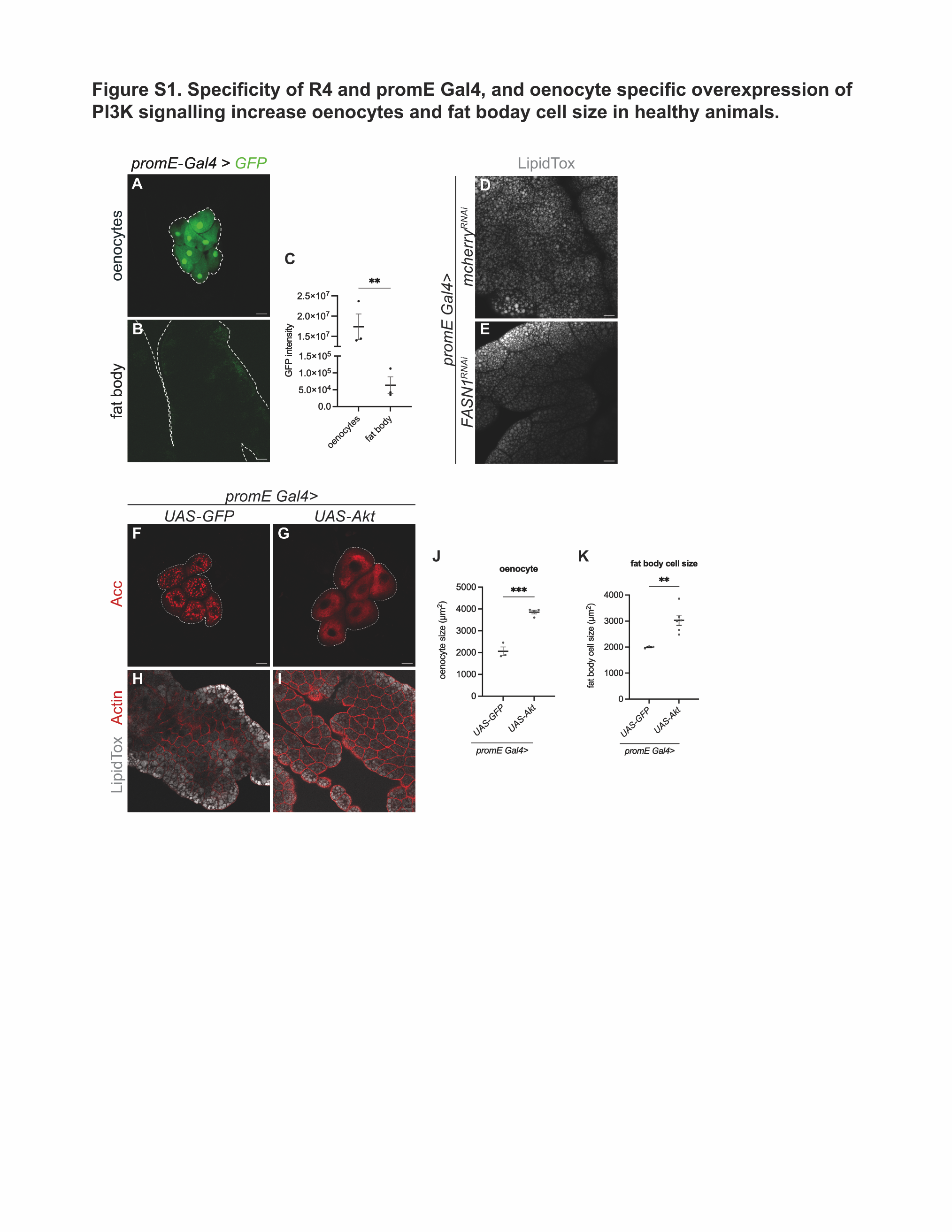

Supplement: S1 Fig — (A-B) Representative image of the oenocytes (A, dashed lines) and fat body (B, dashed lines) from animals with UAS-GFP (green) driven by promE-Gal4. (C) Quantification of GFP intensity, with values averaged across multiple oenocytes (fat body cells) per animal in in (C-D). oenocytes: n = 3, mean ± SEM = 1.737x10^8^ ± 3.156x10^7^. fat body: n = 3, mean ± SEM = 6.360x10^5^ ± 2.486x10^5^. (D-E) Representative maximum projection of the LDs in fat body from healthy animals, where mcherryRNAi (D), FASN1RNAi (E) were expressed in the oenocytes. LipidTox (grey). (F-G) Representative maximum projection of the oenocytes (dashed lines) from healthy animals, where UAS-GFP (F) and UAS-Akt (G) were expressed in the oenocytes. Acc (red). (H-I) Representative maximum projection of the fat body cells from healthy animals, where UAS-GFP (H) and UAS-Akt (I) were expressed in the oenocytes. Fat body stained for phalloidin (Actin) (red), LipidTox (grey). (J) Quantification of oenocyte cell size, with values averaged across multiple oenocytes per animal in (F-G). UAS-GFP: n = 3, mean ± SEM = 2061 ± 198.4. UAS-Akt: n = 4, mean ± SEM = 3855 ± 84.14. (K) Quantification of fat body cell size, with values averaged across multiple fat body cells per animal in (H-I). UAS-GFP: n = 3, mean ± SEM = 1997 ± 24.58. UAS-Akt: n = 6, mean ± SEM = 3032 ± 195.4. Scale bar is 25μm in (A-B, D-G), 50 μm in (H-I). (TIF) [file pgen.1012150.s001.tif]

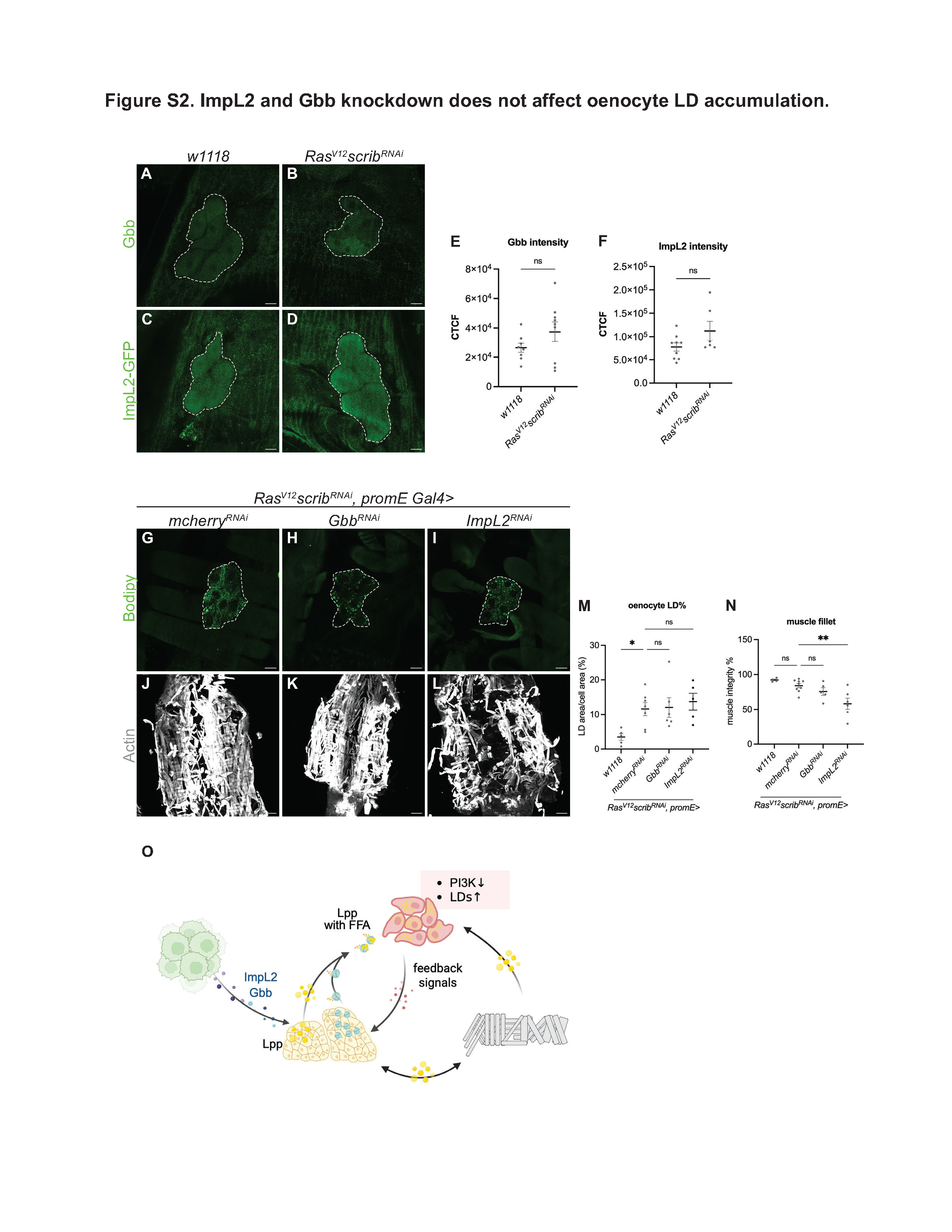

Supplement: S2 Fig — (A-B) Representative maximum projections of the oenocytes (dashed lines) from w1118 (A) and RasV12 scribRNAi tumour-bearing animals (B). Oenocytes stained for Gbb (green). (C-D) Representative maximum projections of the oenocytes (dashed lines) from w1118 (C) and RasV12 scribRNAi tumour-bearing animals (D). Oenocytes expressed endogenous ImpL2-GFP (green). (E) Quantification of Gbb intensity, with values averaged across multiple oenocytes per animal in in (A-B). w1118: n = 8, mean ± SEM = 2.460x10^5^ ± 3132. RasV12 scribRNAi: n = 9, mean ± SEM = 3.736x10^5^ ± 6710. (F) Quantification of ImpL2-GFP intensity, with values averaged across multiple oenocytes per animal in in (C-D). w1118: n = 9, mean ± SEM = 7.714x10^5^ ± 8724. RasV12 scribRNAi: n = 6, mean ± SEM = 11.23x105 ± 2.052x10^5^. (G-I) Representative maximum projections of the oenocytes (dashed lines) from RasV12 scribRNAi tumour-bearing animals, where mcherryRNAi (G), GbbRNAi (H), ImpL2RNAi(I) were expressed in oenocytes. Bodipy (green). (J-L) Representative images of the muscle fillet from Day 6 tumour-bearing animals, where mcherryRNAi (J), GbbRNAi (K), ImpL2RNAi(L) were expressed in the oenocytes. Actin (grey). (M) Quantification of LD area as a percentage of oenocyte cell area, with values averaged across multiple oenocytes per animal in w1118 and (G-I).: n = 5, mean ± SEM = 3.487 ± 0.9776. mcherryRNAi: n = 7, mean ± SEM = 11.56 ± 1.884. GbbRNAi: n = 6, mean ± SEM = 12.02 ± 2.825. ImpL2RNAi: n = 5, mean ± SEM = 13.69 ± 2.434. (N) Quantification of muscle detachment in w1118 and (J-L). w1118: n = 4, mean ± SEM = 92.28 ± 1.234. mcherryRNAi: n = 9, mean ± SEM = 84.09 ± 2.941. GbbRNAi: n = 5, mean ± SEM = 75.71 ± 5.444. ImpL2RNAi: n = 6, mean ± SEM = 58.20 ± 8.011. (O) In cachectic animals, tumours secrete ImpL2 and Gbb, leading to systemic disruption of lipid metabolism. Lipid droplet trafficking occurs dynamically between the fat body, muscle, and oenocytes, with lipid-binding proteins (Lpps) released from the fat b [file pgen.1012150.s002.tif]
