# Supplementary material for: Tumour-driven lipid accumulation in oenocytes reflects systemic lipid alterations
Source: PLoS Genet. 2026 May 7;22(5):e1012150. doi: 10.1371/journal.pgen.1012150 (PMC13167029; doi:10.1371/journal.pgen.1012150)
Supplement: Attachment — Submitted filename: 159449_1_rebuttal_3473710_t0thhc.docx [file pgen.1012150.s003.docx]

**Manuscript number:** RC-2025-03102R

**Corresponding author(s):** Louise Cheng

## 1. General Statements [optional]

*This section is optional. Insert here any general statements you wish to make about the goal of the study or about the reviews.*

## 2. Description of the planned revisions

*Insert here a point-by-point reply that explains what revisions, additional experimentations and analyses are planned to address the points raised by the referees.*

*Answers in Blue*

*Reviewer #1 (Evidence, reproducibility and clarity (Required)): 

Summary 
In this study Liu et al. report that, in larval drosophila models of cachexia, lipids accumulates in oenocytes, specialised cells found along the posterior body of the larva, and which undertake some of the function of mammalian hepatocytes. Using double overexpression systems they then provide evidence that this lipid accumulation in oenocytes during tumour-dependent cachexia might be modulated by lipid synthesis and transport pathways in the Fat Body and Muscles. Conversely, they claim that disrupting oenocytes lipid synthesis alters lipid storage in the Fat body cells mitigating their accumulation during cachexia, but fails to rescue muscle disruption. Molecularly, they show that enhancing Akt activity in oenocytes rescue the accumulation of lipids seen in the presence of tumours which consistently rescues non-autonomously fat body lipid accumulation but not muscle disruption. Finally, the authors report signs of increased Ecdysone and Wg signalling in oenocytes, but fail to show this is functionally required for oenocyte lipid accumulation or cachexia phenotypes in muscles. 


Major Comments 
This study offers a description of the lipid accumulation in oenocytes in larvae with cachexia inducing tumours but several of their main claims are not fully supported by the presented data and need additional work / analyses. 

1. Statistics and oenocyte lipid accumulation. 
From what I could understand from the results, figures, and mat/meth, at least two animals per genotype were analysed... Is that correct? 
So authors plot as n, the number of oenocytes studied. There are several oenocytes per cluster of oenocytes, and several clusters per larvae. So analysing 2 or 3 larvae, probably from the same vial, gives the number of "samples" tested shown on the graphs. Of course, it is highly probable that all oenocytes from the same larvae will behave the same. 
Statistics should thus be done: 
- Take one or two oenocytes clusters per larvae, and count lipid droplets. Make an average. 
- Take then several larvae of the same experiment and make an average 
- Repeat the experiment at least 3 times, and compare then the changes in lipid accumulation in oenocytes. 
Treating each oenocyte cell as an independent sample is inappropriate here.*

*We can represent our data in the form requested by the reviewer.

I am afraid the same is true for fat body lipid droplets size. Each droplet cannot be considered an independent sample. Size should be averaged on different adipocytes from different larvae, and different experiments (ie vials where the cross is repeated) should be compared.*

*We can represent the fat body lipid droplet data as suggested by the reviewer.

The authors approach falsely increases their experimental n, and thus lowers artificially the p-value of their statistical tests.*

*2. A cachexia phenotype? 
Authors claim the lipid accumulation in oenocytes is a cachexia-associated phenotype. But they only compare wild-type larvae with two models of larvae bearing cachexia-inducing tumours which are developmentally delayed. They should thus show what happens to oenocytes in animals with tumours that do not induce cachexia which are also developmentally delayed. 
Only then can they dismiss the effect of developmental delay, and claim that this is truly a phenotype associated with cachexia.*

*We already have data at hand, where we have shown in developmentally delayed animals caused by neuroblast tumour induced via ProsRNAi, which we have previously shown to not cause cachexia (Lodge et al., 2021, Dev Cell), does not cause lipid droplet accumulation phenotype. Thus, the phenotype presented is highly specific to cachexia.

3. Tumour burden. 
Using elegant double systems, authors manipulate the fat body and muscles lipid metabolism and then question whether this will affect lipid droplets accumulation in oenocytes. However it should be noted that the drivers they use (R4 or Mhc-Gal4 for the fat body and muscles respectively) start expression from the beginning of larval life, as soon as, or earlier than tumour initiation. This is particularly relevant for the fat body, that through its endocrine function, is a major regulator of organismal growth and general metabolism. 
So for all the experiments where the oenocytes, muscles, and critically the fat body are manipulated, authors must show the tumour growth and that tumour burden is not affected. 
Only if tumour burden is not reduced, can they support the interorgan circuits they claim take place during cachexia between oenocytes, fat body, and muscles, instead of an indirect effect due to reduced tumour growth (and thus lower release of tumour derived cachectic factors).*

*We will measure tumour size in all genetic manipulations as suggested by the reviewer.  

4. Lipid accumulation in Fat body cells and cachexia 
The size of lipid droplets is clearly affected during cachexia. However, it should be noted that there is a big variability in the images shown using lipidtox (compare Fig. 2A with 4D). I am afraid that some of the phenotypes shown here might be showing extremely sick adipocytes. An actin staining at the same time showing adipocyte outline would help.*

*We will present data with co-staining of actin staining as suggested.*

*Authors should also measure adipocyte cell size, which has been shown to decrease during cachexia.*

*We will measure adipocyte size as suggested by the reviewer.*

*In parallel to lipid droplets accumulation, authors should measure TAG content in Fat bodies to firmly establish the claim that lipid accumulates in adipocytes during cachexia.*

*We will present TAG measurement in the fat body as suggested.

5. Image acquisition and quantification 
In several panels, it is hard to understand why the background is more intense, undermining some of the quantifications being presented: 
Bodipy in oenocytes: Figure 3G compared to 3E and 3F, 5F' compared to 5I', and to a lesser extend 4B compared to 4E.*

*The staining in oenocytes is more variable than other tissues, because it is buried under muscles. The data we present is always presented with the control that is acquired under the same confocal setting. On different days, depending on the sample, the staining intensity can differ, but as control is always shown in parallel, we believe we have presented fair comparisons.*

*Similar comments for FOXO-GFP between 5B and 5C. Why is there more FOXO-GFP everywhere?*

*We do see more FOXO-GFP outside of the nucleus, thus we will measure nuclear cytoplasmic ratio.*

*Could authors use another readout of Insulin signalling such as InR expression?*

*As isolating oenocytes is not possible, we can not measure InR expression.*

*Or tGPH localisation?*

*We will perform tGPH as suggested.*

*Specifically regarding FOXO, its activity is regulated by its nuclear import. I would like to see a quantification of nucleus/cytoplasm quantification rather than nuclear intensity normalised by DAPI as is currently shown, which fails to normalise background levels between different slides / experiments.*

*We will present nuclear/cytoplasmic ratio as suggested.

6. Ecdysone and Wg signalling. 
The data presented here is not really convincing. Here again, there are issues with different background levels (see S2A and S2B).*

*Background difficult to avoid, due to where the oenocytes are located.*

*Why do authors focus on nkd-LacZ in the nucleus? Does the nkd-LacZ transgene carry a nuclear localisation sequence? There are no details in the M&M.*

*Yes, there is a nuclear signal, we will amend the Material and Methods.*

*As clear with DAPI size, the animals shown are not of similar age (larger nuclei in the Ras dlg). Here it would be informative to 
- Either look earlier at day 5 when Ras dlg animals are of similar age as wild type controls (maybe a difference already exists)*

*We will perform this experiment using RasScrib animals at day 5-6 and compare with WT at day 5-6, so they are more comparable.*

*- Take as control animals with cachexia non-inducing tumours but delayed animals (see point 2)*

*As the genotype of QF2 elav; QUAS-Pros RNAi animals is already complex, it would be difficult to assess the expression of nkd-lacZ in these animals, thus we will try option 1.*

*For Ecdysone, it is unclear how animals would get higher ecdysone signalling? The tumour bearing animals are clearly developmentally delayed, consistent with lower ecdysone levels. Santabárbara-Ruiz & Léopold published that in the same model of Ras dlg tumours in the eye disc, tumours represent a sink for ecdysone. Comparing ecdysone signalling is extremely sensitive to developmental stage, for instance whether the spiracle are everted or not on L3 wandering larvae...). Since cachectic larvae are delayed, this will require extremely tight controls. Again the control of delayed animals with cachexia non-inducing tumours would be great.*

*We will try Brd staining in RasScrib animals at day 5-6 and compare with elav QF2>QUAS prosRNAi animals.*

*Authors should also explore in more details ecdysone signalling using other readouts besides Broad protein levels, such as transcriptional expression levels of Ecdysone signalling when possible (qPCR for Ecdysone targets on oenocytes might be too complicated, but specific LacZ reporters could be used).*

*We will try using a EcR-GFP as an alternative readout.*

*As a side note, authors claim in the text in line 277-279 that they have used an ecdysone-inducible reporter to monitor Ecdysone signalling, but on the figure S2 and M&M, it is clear they have used an anti-Broad immune-staining.*

*We will correct this mistake.*

*The fact that impairing either wg or Ecdysone signalling in oenocytes does not modify the phenotypes further undermines the up-regulation of these pathways in oenocytes (even though it is entirely possible that their upregulation is not functionally participating to the lipid accumulation). 

7. The role of oenocytes during cachexia 
Authors should discuss, given that disrupting lipid metabolism and preventing lipid droplets enlargement in fat body cells rescues muscle disruption, why lipid metabolism disruption in oenocytes, which impacts lipid metabolism in fat body cells and prevents lipid droplets enlargement, does not then translate in a rescue of muscle disruption? 
we will discuss this.


Minor Comments 
1. Action of Gbb and ImpL2 
In Figure 1, authors show a diagram where tumour derived Gbb and ImpL2 act directly on oenocyte cells. This is not backed by the data as the authors have not directly tested this. Moreover previous studies, including from this lab, showed that tumour-derived ImpL2 and Gbb act on Fat body. So authors should change the diagram and not imply that the Gbb and ImpL2 act directly on the oenocytes.*

*we will change the diagram.

2. Why is there more Acc in oenocytes in the Ras-Scrib than in the Ras-Dlg model? Is that just variability in staining between different larvae?*

*This is just variability in staining. We have a quantification for Acc in all the genotypes, and it was totally randomized.

3. Source of lipids in oenocytes 
If I understood correctly, oenocytes need both de-novo synthesis AND transport from the fat body AND transport from muscles for lipid droplet accumulation? Authors should discuss though why there is no redundancy between these three sources?*

*We will discuss this.

4. RNAi efficacy should be shown, in particular for the RNAi lines which did not show any changes in phenotypes (e.g. pan-RNAi)*

*The efficiency of pan-RNAi was published, which we have cited* (Zhang *et al*, 2017).

*5. Authors should discuss how their work on the exchange network between oenocytes, fat body, and muscles is different in the presence of tumours in comparison to wild type larvae.*

*We can discuss

6. Authors should indicate clearly on each panel the age of the images shown (days post AEL) so the reader could easily grasp whether rescued phenotypes are studied at similar developmental delay or not (a key aspect in the understanding of the system). 
We will amend.

Reviewer #1 (Significance (Required)): 

Significance 
While the strength of this study resides in that it addresses oenocytes, a cell type that has not benefited from a lot of attention thus far in the context of tumour development, and uses refined genetic tools (double systems), it presents major weaknesses. Many of the claims presented are not robustly supported by the experiments shown with issues with statistics and sample definition, with image quantification, or by the lack of normalisation by tumour burden, which represents an obvious confounding factor that is never addressed. 
This study is for a specialised audience and the link between the disruption observed in oenocytes (drosophila) and hepatocytes (mammals) during cachexia, while used in the introduction as a rationale for the study, is here not convincing, since the effect of cachexia non-inducing tumours has not been studied. 

Expertise in Drosophila development 


Reviewer #2 (Evidence, reproducibility and clarity (Required)): 

Liu et al report on lipid accumulation in oenocytes in tumour-bearing fly larva. By using the power of Drosophila genetics, they identify a number of factors and pathways that control the lipid accumulation, Overall, lipid accumulation in oenocytes seems to be downstream of fat body lipolysis, which is, for example, under the control of the insulin antagonist, ImpL2, secreted from the tumour. In the oenocytes themselves, de novo lipogenesis also seems to play a role for lipid accumulation. 
The findings are interesting and the experiments are well executed. All conclusions are supported by the results. In my opinion, the paper could improve by showing the importance of the lipid accumulation for animal survival. This could be done by performing more functional health tests, incl. scoring developmental progression or, even, survival. I also think that comparisons between normal and tumour-bearing animals are generally missing in the paper. 


Major comments: 
1. in Fig. 1, the knockdowns of Impl2 and Gbb suggest that the two tumour-secreted factors function in parallel to affect oenocyte lipid accumulation during cancer cachexia. How specific to the tumor is this effect? What happens to the lipid accumulation if ImpL2 and Gbb is silenced in oenocytes in the QUAS RasV12, scrib RNAi tumor model?*

*We will look at whether Gbb or ImpL2 is upregulated in oenocytes first, and if upregulated we will knock them down in the oenocytes, however, the double knockdown of these ligands will not be possible due to the limitation of the genetic system.*

*2. Why is ACC used as a marker for LDs if on day 5 ACC is already upregulated while LDs do not appear before day 6?*

*Acc is used to mark oenocytes, we use bodipy to mark lipid droplets.*

*3. Typically, the insulin pathway promotes lipid and carbohydrate storage, but here the suppression of ImpL2 secretion from the tumour as well as AKT expression in oenocytes leads to less lipid storage oenocytes. Please comment on this discrepancy with the literature.*

*Our observation is consistent with published literature, which we have discussed in our discussion section.*

*4. As lipolysis is under the control of Bmm, expression Bmm should be studied.*

*We will present Bmm qPCR data.*

*The effect of Bmm KD in FB for lipid accumulation in oenocytes should also be studied.*

*We have done this manipulation and will present the data.*

*5. Please describe in more detail how muscle integrity is assessed (Fig. 2N)*

*We have referenced the methods paper (Dark et al., 2022), and will include more details in the M&M.*

*6. „, these results indicate that disrupting lipid synthesis and/or storage in the fat body is sufficient to suppress oenocyte lipid accumulation in cachectic animals". Is this only true for cachectic animals?*

*WT animals do not accumulate lipid droplets, thus it is difficult to assess if this is true only for cachectic animals. It has been published before some of these effects under nutrient restriction, we will discuss this.*

*7. Fig. 5C: why is Foxo staining increased outside the oenocytes?*

*We willl look at other readouts of PI3K, and also present the Foxo data as nuclear/cytoplasmic ratio.*

*8. What does lipid accumulation in oenocytes mean for animal fitness during cancer cachexia? Please add some experiments.*

*We will do a pupariation assay to examine the relationship between muscle integrity and survival, and thus infer the relationship between lipid accumulation/muscle integrity and survival.*

*9. Please add comparisons between normal and tumour-bearing animals throughout the paper. 
We will add WT control to compare with tumour bearing data.

Minor comments: 
1. Please include scale bar in all images. (e.g., Figure1 B-E, Figure2 C-E) 
2. In Figure2Q-R, what does ApoIII stand for? 
3. Please incude nuclear staining in the Figure2Q and R, they do not seem to be in the same layer. 
4. Please change the order of supplement figures according to the text. 
We will fix these minor corrections.


Reviewer #2 (Significance (Required)): 

If the points above are addressed, the significance is high. In my opinion, it will be important to be less descriptive. While understanding the pathways regulating lipid accumulation in oenocytes is interesting, it would be much more appealing to understand why lipid accumulation occurs and what it means for animal fitness during cancer cachexia. 


Reviewer #3 (Evidence, reproducibility and clarity (Required)): 

Summary: 
In this study, the authors use Drosophila larvae to investigate how epithelial tumors induce systemic metabolic changes characteristic of cancer cachexia. They focus on oenocytes-hepatocyte-like cells-and demonstrate that these cells accumulate lipid droplets in response to tumor-secreted factors Gbb and ImpL2. This lipid accumulation is modulated by metabolic alterations in distant tissues, including the fat body and muscle, revealing bidirectional non-autonomous crosstalk. The authors employ Gal4/UAS and QF/QUAS binary systems to independently manipulate tumors and target tissues. They show that changes in lipid synthesis, storage, or transport in the fat body or muscle influence oenocyte lipid content, and that oenocyte-specific manipulations can reciprocally affect fat body lipid stores. However, these oenocyte lipid changes do not influence muscle wasting, indicating that oenocytes are not central to muscle integrity during cachexia. The study also implicates reduced PI3K in oenocytes of tumor-bearing larvae as a regulator of oenocyte lipid levels. Overall, the work provides a nice view of inter-organ metabolic communication during tumor-induced wasting, showing how oenocytes are important modulators of systemic lipid status in tumor-bearing animals. 

Major comments: 
Overall this is a very well performed piece of work. The data are all well-presented and convincing, and the methods are clearly presented to allow other researchers to build on this work. Also, all the experiments adequately replicated and statistical analysis are excellent 

My only main comment concerns the result in Fig 5 showing increased FOXO in oenocytes of tumor bearing. The authors use this result to argue that these oenocytes have lower PI3/Akt activity. However, I think this result could be strengthened - FOXO is typically regulated by PI3k/AKT through control of its nuclear localization, ie mostly cytoplasmic when pI3k/Akt activity is high and strongly nuclear when PI3K/Akt activity is inhibited. In this context, the FOX-GFP results in Fig 5C are a little tricky to interpret. The main effect seems to be a general overall increase in the FOX)-GFP levels in the tumor-bearing larvae rather than any change in foxo nuclear localization, in which case the result is difficult to interpret as a downregulation of PI3k/Akt. 

Can the authors clarify how they quantified this result? A higher magnification image without the added outlines of the nuclei would be helpful to interpret the result.*

*Can they/did they look at cytoplasmic:nuclear staining of FOXO-GFP? 
We will redo this experiment and present the data as cytoplasmic:nuclear ratio.

The interpretation of this result would be strengthened with other additional measures of Pi3/Akt/FOXO signaling such as 1) direct staining of Foxo using a Foxo antibody (this isn't commercially available but is available from labs who work on Foxo)*

*or 2) staining for thor-LacZ - thor is a foxo target and so expression could be higher in the oenocytes from tumor animals,*

*or 3) the tGPH reporter (Britton et al 2003, Dev Cell). 
We will try foxo staining, thor-lacZ and tGPH.*

*These additional experiments should take 2-3 months. 

Minor comments: 

- The introduction would benefit from a little more background on previous Drosophila studies looking at how disc tumors affect systemic lipid metabolism and fat/muscle wasting. This would help provide a little more context about the current work. 
- Line 150. Should there be a sentence or explanation before, "To test this..." (to explain hat 'this' refers to 
- Line 219 needs a reference to the relevant figure/panels. 


Reviewer #3 (Significance (Required)): 

Overall, the work makes an important conceptual addition to the field of tumor-host interactions. It builds upon previous work showing bi-directional communication between oenocytes and fat cells to show how these interactions operate in the context of a tumor model to control systemic lipid metabolism. Hence, it provides new insight into our knowledge of how tumor-host interactions modulate lipid biology. 

The primary factor that constrains the conceptual impact of the work is that manipulating oenocyte lipid metabolism didn't alter the overall cachectic phenotype. In this respect, the work, while excellent, doesn't provide as substantial a conceptual advance as other key papers in the field (including important and elegant work from the Cheng lab - e.g., Lodge et al Dev Cell 2021, Dark et al EMBO Rep 2024) that offer deeper insight into the signaling pathways and cellular mechanisms by which tumors affect both fat and muscle biology to induce tissue wasting. This limitation arises not from any shortcoming in the work or methodology, but simply reflects that the biological processes uncovered do not directly connect to cachexia regulation. 

This work would be of interest to a number to fly researchers examining systemic physiology and metabolism and cancer researchers studying host-tumor interactions, as well as researchers generally interested in inter-organ communication and systemic lipid metabolism. Hence the work would be of broad interest. 

My area of expertise is in using Drosophila to study oncogene/tumor suppressor signaling pathways and their effects on growth and systemic physiology.*

## 3. Description of the revisions that have already been incorporated in the transferred manuscript

*Please insert a point-by-point reply describing the revisions that were already carried out and included in the transferred manuscript. If no revisions have been carried out yet, please leave this section empty.*

## 4. Description of analyses that authors prefer not to carry out

*Please include a point-by-point response explaining why some of the requested data or additional analyses might not be necessary or cannot be provided within the scope of a revision. This can be due to time or resource limitations or in case of disagreement about the necessity of such additional data given the scope of the study. Please leave empty if not applicable.*

*Review 1, point 6:*

*- Take as control animals with cachexia non-inducing tumours but delayed animals (see point 2)*

*As the genotype of QF2 elav; QUAS-Pros RNAi animals is already complex, it would be difficult to assess the expression of nkd-lacZ in these animals, thus we will try option 1.*

*Reviewer 2 point 1:*

*1. in Fig. 1, the knockdowns of Impl2 and Gbb suggest that the two tumour-secreted factors function in parallel to affect oenocyte lipid accumulation during cancer cachexia. How specific to the tumor is this effect? What happens to the lipid accumulation if ImpL2 and Gbb is silenced in oenocytes in the QUAS RasV12, scrib RNAi tumor model?*

*We will look at whether Gbb or ImpL2 is upregulated in oenocytes first, and if upregulated we will knock them down in the oenocytes, however, the double knockdown of these ligands will not be possible due to the limitation of the genetic system.*
