# Supplementary material for: Tumour-driven lipid accumulation in oenocytes reflects systemic lipid alterations
Source: PLoS Genet. 2026 May 7;22(5):e1012150. doi: 10.1371/journal.pgen.1012150 (PMC13167029; doi:10.1371/journal.pgen.1012150)
Supplement: Attachment — Submitted filename: full-revision Liu et al.docx [file pgen.1012150.s004.docx]

**Manuscript number:** RC-2025-03102R

**Corresponding author(s):** Louise Cheng

## 1. General Statements [optional]

*In this revision, we have added new data to quantify fat body cell size, tumour size and pupariation rate for all our genetic manipulations. We have performed a new experiment to examine whether PI3K is altered in tumour bearing animals using tGPH. We also explored if ImpL2 or gbb are upregulated in the oenocytes of tumour bearing animals, and if they are functionally involved in lipid metabolism and muscle integrity. We have added Bmm data and withdrew dgat1 data, as the dgat1 data is going into a different paper in preparation, thus we could not provide the full details requested by the reviewer. Due to the inability to demonstrate that wnt/wingless and ecdysone signalling is reproducibly upregulated, we have withdrawn these data and have modified our conclusions accordingly.*

## 2. Point-by-point description of the revisions

*Answers in Blue*

*Reviewer #1 (Evidence, reproducibility and clarity (Required)):* 

*Summary* 
*In this study Liu et al. report that, in larval drosophila models of cachexia, lipids accumulate in oenocytes, specialised cells found along the posterior body of the larva, and which undertake some of the function of mammalian hepatocytes. Using double overexpression systems, they then provide evidence that this lipid accumulation in oenocytes during tumour-dependent cachexia might be modulated by lipid synthesis and transport pathways in the Fat Body and Muscles. Conversely, they claim that disrupting oenocytes lipid synthesis alters lipid storage in the Fat body cells mitigating their accumulation during cachexia but fails to rescue muscle disruption. Molecularly, they show that enhancing Akt activity in oenocytes rescue the accumulation of lipids seen in the presence of tumours which consistently rescues non-autonomously fat body lipid accumulation but not muscle disruption. Finally, the authors report signs of increased Ecdysone and Wg signalling in oenocytes but fail to show this is functionally required for oenocyte lipid accumulation or cachexia phenotypes in muscles.* 


*Major Comments* 
*This study offers a description of the lipid accumulation in oenocytes in larvae with cachexia inducing tumours but several of their main claims are not fully supported by the presented data and need additional work / analyses.* 

*1. Statistics and oenocyte lipid accumulation.* 
*From what I could understand from the results, figures, and mat/meth, at least two animals per genotype were analysed... Is that correct?*

*At least three animals were analyzed per genotype.*

*So, authors plot as n, the number of oenocytes studied. There are several oenocytes per cluster of oenocytes, and several clusters per larvae. So, analyzing 2 or 3 larvae, probably from the same vial, gives the number of "samples" tested shown on the graphs. Of course, it is highly probable that all oenocytes from the same larvae will behave the same.* 
*Statistics should thus be done:* 
*- Take one or two oenocytes clusters per larvae, and count lipid droplets. Make an average.* 
*- Take then several larvae of the same experiment and make an average* 
*- Repeat the experiment at least 3 times and compare then the changes in lipid accumulation in oenocytes.* 
*Treating each oenocyte cell as an independent sample is inappropriate here.*

*We have represented our data in the form requested by the reviewer.*

*I am afraid the same is true for fat body lipid droplets size. Each droplet cannot be considered an independent sample. Size should be averaged on different adipocytes from different larvae, and different experiments (i.e. vials where the cross is repeated) should be compared.*

*We have represented our data in the form requested by the reviewer*

*The authors approach falsely increases their experimental n and thus lowers artificially the p-value of their statistical tests.*

*2. A cachexia phenotype?* 
*Authors claim the lipid accumulation in oenocytes is a cachexia-associated phenotype. But they only compare wild-type larvae with two models of larvae bearing cachexia-inducing tumours which are developmentally delayed. They should thus show what happens to oenocytes in animals with tumours that do not induce cachexia which are also developmentally delayed.* 
*Only then can they dismiss the effect of developmental delay and claim that this is truly a phenotype associated with cachexia.*

*Neuroblast tumours induced via ProsRNAi does not cause cachexia, [1]) but causes developmental delay ([2], Figure reproduced on the left as Fig R1). We examined in these animals, whether oenocytes accumulated lipid droplets (Figure 1). We found that the oenocyte lipid accumulation phenotype to be highly specific to cachexia and does not occur in QF-QprosRNAi tumour bearing animals.*

*Fig R1: elavQF>QprosRNAi tumour-bearing animals exhibit delayed pupariation compared to elavQF>+ control. Statistical analysis performed using a two-way ANOVA followed by Šídák’s multiple comparisons post-hoc test.*

*3. Tumour burden.* 
*Using elegant double systems, authors manipulate the fat body and muscles lipid metabolism and then question whether this will affect lipid droplets accumulation in oenocytes. However, it should be noted that the drivers they use (R4 or Mhc-Gal4 for the fat body and muscles respectively) start expression from the beginning of larval life, as soon as, or earlier than tumour initiation. This is particularly relevant for the fat body, that through its endocrine function, is a major regulator of organismal growth and general metabolism.* 
*So, for all the experiments where the oenocytes, muscles, and critically the fat body are manipulated, authors must show the tumour growth and that tumour burden is not affected.* 
*Only if tumour burden is not reduced, can they support the interorgan circuits they claim take place during cachexia between oenocytes, fat body, and muscles, instead of an indirect effect due to reduced tumour growth (and thus lower release of tumour derived cachectic factors).*

*We have measured tumour size in all the genetic manipulations as suggested by the reviewer.*

*4. Lipid accumulation in Fat body cells and cachexia* 
*The size of lipid droplets is clearly affected during cachexia. However, it should be noted that there is a big variability in the images shown using lipidtox (compare Fig. 2A with 4D). I am afraid that some of the phenotypes shown here might be showing extremely sick adipocytes. An actin staining at the same time showing adipocyte outline would help.*

*We have presented all the fat body images with co-staining of actin.*

*Authors should also measure adipocyte cell size, which has been shown to decrease during cachexia.*

*We have measured fat body cell size as suggested by the reviewer.*

*In parallel to lipid droplets accumulation, authors should measure TAG content in Fat bodies to firmly establish the claim that lipid accumulates in adipocytes during cachexia.*

*Despite lipid droplets enlargement phenotype, we believe the phenotype is due to lipid coalescence, and not an increase in TAG, this is the focus of another manuscript under preparation. We have reproduced the TAG data and present it as Fig R2.*

*Figure R2: Lipidomics was performed on 5 replicas of control (R4>) or tumour (Ras^v12^scrib^Ri^;R4>mCherry^Ri^) fat body at 6 days AEL. Statistical analysis performed using student t-test (*, p<0.01)*

*5. Image acquisition and quantification* 
*In several panels, it is hard to understand why the background is more intense, undermining some of the quantifications being presented:* 
*Bodipy in oenocytes: Figure 3G compared to 3E and 3F, 5F' compared to 5I', and to a lesser extend 4B compared to 4E.*

*The staining in oenocytes is more variable than other tissues, because it is buried under muscles. The data we present is always presented with the control that is acquired under the same confocal setting. The background staining is due to lipid staining in surround tissues such as the muscle, which we have previously shown to accumulate lipids upon systemic lipid manipulations [3].*

*Similar comments for FOXO-GFP between 5B and 5C. Why is there more FOXO-GFP everywhere?*

*We do see more FOXO-GFP outside of the nucleus, we have now presented our data as nuclear cytoplasmic ratio.*

*Could authors use another readout of Insulin signalling such as InR expression?*

*Or tGPH localisation?*

*We have performed tGPH experiments, which are presented in Fig 5.*

*Specifically regarding FOXO, its activity is regulated by its nuclear import. I would like to see a quantification of nucleus/cytoplasm quantification rather than nuclear intensity normalised by DAPI as is currently shown, which fails to normalise background levels between different slides / experiments.*

*We have presented FOXO data as nuclear/cytoplasmic ratio as suggested by the reviewer in Figure 5.*

*6. Ecdysone and Wg signalling.*

*For Wg and ecdysone signaling section, we have repeated the experiments with controls suggested by the reviewer and have found indeed the staining is variable and ecdysone staining fluctuates with developmental stages. Thus, we can no longer conclude that Wg or ecdysone signaling is upregulated in the oenocytes and have withdrawn this data from Figure S2.*

**We have repeated nkz-lacZ staining and have found that the staining is more variable than we initially observed, and we could not see a difference between control and tumour.*

** For ecdysone readout, we tried assessing Broad staining in developmentally delayed, non-cachectic animals (ElavQF>prosRi) and found Broad staining is variable according to developmental stages. We did not see an upregulation of Broad as we initially found, thus we have withdrawn our data on ecdysone signaling.*

*The data presented here is not really convincing. Here again, there are issues with different background levels (see S2A and S2B).*

*Why do authors focus on nkd-LacZ in the nucleus? Does the nkd-LacZ transgene carry a nuclear localisation sequence? There are no details in the M&M.*

*As clear with DAPI size, the animals shown are not of similar age (larger nuclei in the Ras dlg). Here it would be informative to* 
*- Either look earlier at day 5 when Ras dlg animals are of similar age as wild type controls (maybe a difference already exists)*

*- Take as control animals with cachexia non-inducing tumours but delayed animals (see point 2)*

*The related data was withdrawn from the manuscript*

*For Ecdysone, it is unclear how animals would get higher ecdysone signalling? The tumour bearing animals are clearly developmentally delayed, consistent with lower ecdysone levels. Santabárbara-Ruiz & Léopold published that in the same model of Ras dlg tumours in the eye disc, tumours represent a sink for ecdysone. Comparing ecdysone signalling is extremely sensitive to developmental stage, for instance whether the spiracle are everted or not on L3 wandering larvae...). Since cachectic larvae are delayed, this will require extremely tight controls. Again, the control of delayed animals with cachexia non-inducing tumours would be great.*

*The related data was withdrawn from the manuscript*

*Authors should also explore in more details ecdysone signalling using other readouts besides Broad protein levels, such as transcriptional expression levels of Ecdysone signalling when possible (qPCR for Ecdysone targets on oenocytes might be too complicated, but specific LacZ reporters could be used).*

*The related data was withdrawn from the manuscript*

*As a side note, authors claim in the text in line 277-279 that they have used an ecdysone-inducible reporter to monitor Ecdysone signalling, but on the figure S2 and M&M, it is clear they have used an anti-Broad immune-staining.*

*The related data was withdrawn from the manuscript*

*The fact that impairing either wg or Ecdysone signalling in oenocytes does not modify the phenotypes further undermines the up-regulation of these pathways in oenocytes (even though it is entirely possible that their upregulation is not functionally participating to the lipid accumulation).*

*The related data was withdrawn from the manuscript*

*7. The role of oenocytes during cachexia* 
*Authors should discuss, given that disrupting lipid metabolism and preventing lipid droplets enlargement in fat body cells rescues muscle disruption, why lipid metabolism disruption in oenocytes, which impacts lipid metabolism in fat body cells and prevents lipid droplets enlargement, does not then translate in a rescue of muscle disruption?* 
*we have added this to our discussion.*


*Minor Comments* 
*1. Action of Gbb and ImpL2* 
*In Figure 1, authors show a diagram where tumour derived Gbb and ImpL2 act directly on oenocyte cells. This is not backed by the data as the authors have not directly tested this. Moreover previous studies, including from this lab, showed that tumour-derived ImpL2 and Gbb act on Fat body. So authors should change the diagram and not imply that the Gbb and ImpL2 act directly on the oenocytes.*

*we have changed the diagram.*

*2. Why is there more Acc in oenocytes in the Ras-Scrib than in the Ras-Dlg model? Is that just variability in staining between different larvae?*

*Acc intensity in oenocytes of different genotypes showed that Acc levels are variable (Figure R4), and this is not dependent on the tumour genotype.*

*Figure R4: quantification of Acc intensity in oenocytes under genetic manipulations in tumour bearing animals.*

*3. Source of lipids in oenocytes* 
*If I understood correctly, oenocytes need both de-novo synthesis AND transport from the fat body AND transport from muscles for lipid droplet accumulation? Authors should discuss though why there is no redundancy between these three sources?*

*We have discussed this.*

*4. RNAi efficacy should be shown, in particular for the RNAi lines which did not show any changes in phenotypes (e.g. pan-RNAi)*

*Due to the inconsistency of our reporter expression for nkz-lacZ in oenocytes, we have withdrawn the wnt readouts and pan RNAi data.*

*5. Authors should discuss how their work on the exchange network between oenocytes, fat body, and muscles is different in the presence of tumours in comparison to wild type larvae.*

*We have added this to our discussion.*

*6. Authors should indicate clearly on each panel the age of the images shown (days post AEL) so the reader could easily grasp whether rescued phenotypes are studied at similar developmental delay or not (a key aspect in the understanding of the system).* 
*We have added this information to the figure legend.*

*Reviewer #1 (Significance (Required)):* 

*Significance* 
*While the strength of this study resides in that it addresses oenocytes, a cell type that has not benefited from a lot of attention thus far in the context of tumour development, and uses refined genetic tools (double systems), it presents major weaknesses. Many of the claims presented are not robustly supported by the experiments shown with issues with statistics and sample definition, with image quantification, or by the lack of normalisation by tumour burden, which represents an obvious confounding factor that is never addressed.* 
*This study is for a specialised audience and the link between the disruption observed in oenocytes (drosophila) and hepatocytes (mammals) during cachexia, while used in the introduction as a rationale for the study, is here not convincing, since the effect of cachexia non-inducing tumours has not been studied.* 

*Expertise in Drosophila development* 


*Reviewer #2 (Evidence, reproducibility and clarity (Required)):* 

*Liu et al report on lipid accumulation in oenocytes in tumour-bearing fly larva. By using the power of Drosophila genetics, they identify a number of factors and pathways that control the lipid accumulation, Overall, lipid accumulation in oenocytes seems to be downstream of fat body lipolysis, which is, for example, under the control of the insulin antagonist, ImpL2, secreted from the tumour. In the oenocytes themselves, de novo lipogenesis also seems to play a role for lipid accumulation.* 
*The findings are interesting and the experiments are well executed. All conclusions are supported by the results. In my opinion, the paper could improve by showing the importance of the lipid accumulation for animal survival. This could be done by performing more functional health tests, incl. scoring developmental progression or, even, survival. I also think that comparisons between normal and tumour-bearing animals are generally missing in the paper.* 


*Major comments:* 
*1. in Fig. 1, the knockdowns of Impl2 and Gbb suggest that the two tumour-secreted factors function in parallel to affect oenocyte lipid accumulation during cancer cachexia. How specific to the tumor is this effect? What happens to the lipid accumulation if ImpL2 and Gbb is silenced in oenocytes in the QUAS RasV12, scrib RNAi tumor model?*

*We have examined whether Gbb or ImpL2 is upregulated in oenocytes, we found a trend towards upregulation. Thus, we performed new experiments to knock them down in the oenocytes and found that while these manipulations did not have an effect on lipid accumulation in oenocytes, ImpL2 knockdown did significantly worsen muscle disruption phenotype (Figure 6).*

*2. Why is ACC used as a marker for LDs if on day 5 ACC is already upregulated while LDs do not appear before day 6?*

*Acc is used to mark oenocytes as shown previously in Cinnamon et al., 2016, its levels are variable across different genotypes, we do not think it is linked to function (Figure R4).*

*3. Typically, the insulin pathway promotes lipid and carbohydrate storage, but here the suppression of ImpL2 secretion from the tumour as well as AKT expression in oenocytes leads to less lipid storage oenocytes. Please comment on this discrepancy with the literature.*

*In Cinnamon et al., 2016, in WT larval oenocytes [4], it was shown that inhibition of PI3K siganlling causes accumulation of lipid droplets during nutrient restriction, and this accumulation is inhibited by the activation of PI3K.*

*Oenocyte-specific inhibition of akt in the adult [5] also caused an accumulation of lipid droplets.*

*Both of these papers are consistent with our data, where we found that in tumour bearing animals, we observed a downregulation of PI3K in the oenocytes, and an accumulation of lipid droplets. The activation of AKT in the oenocytes of tumour bearing animals, or inhibition of ImpL2 from the tumour (which in turn activates PI3K peripherally), reduces lipid accumulation.*

*4. As lipolysis is under the control of Bmm, expression Bmm should be studied.*

*We have presented Bmm qPCR data in Figure 2.*

*The effect of Bmm KD in FB for lipid accumulation in oenocytes should also be studied.*

*We have done this manipulation in Figure 2.*

*5. Please describe in more detail how muscle integrity is assessed (Fig. 2N)*

*We have referenced the methods paper and added more details (Dark et al., 2022).*

*6. „, these results indicate that disrupting lipid synthesis and/or storage in the fat body is sufficient to suppress oenocyte lipid accumulation in cachectic animals". Is this only true for cachectic animals?*

*WT animals do not accumulate lipid droplets. In Cinnamon et al., 2016, it has been shown that the expression of RNAis against Dgat1, and Acc in the oenocytes caused decreased or blocked induction of lipid droplets under nutrient restriction. It would be interesting to test if under NR, fat body manipulations of these pathways can alter oenocyte lipid accumulation.*

*7. Fig. 5C: why is Foxo staining increased outside the oenocytes?*

*We have presented the Foxo data as nuclear/cytoplasmic ratio, to take into account that some FOXO is found outside of the nucleus.*

*8. What does lipid accumulation in oenocytes mean for animal fitness during cancer cachexia? Please add some experiments.*

*Muscle integrity is a good proxy for animal survival. We have added muscle integrity data for fatbody specific Bmm RNAi manipulation, and have added pupariation assays to further strengthen our argument that when we see no muscle rescue, animal survival is not enhanced, but when we see rescue of muscle integrity, animal survival is enhanced.*

*9. Please add comparisons between normal and tumour-bearing animals throughout the paper.* 
*We have added WT control to compare with tumour bearing data throughout the manuscript.*

*Minor comments:* 
*1. Please include scale bar in all images. (e.g., Figure1 B-E, Figure2 C-E)*

*Done*
*2. In Figure2Q-R, what does ApoIII stand for?*

*Apolipoprotein II.* 
*3. Please include nuclear staining in the Figure2Q and R, they do not seem to be in the same layer.*

We have added this.
*4. Please change the order of supplement figures according to the text.* 
*We have fixed this.*


*Reviewer #2 (Significance (Required)):* 

*If the points above are addressed, the significance is high. In my opinion, it will be important to be less descriptive. While understanding the pathways regulating lipid accumulation in oenocytes is interesting, it would be much more appealing to understand why lipid accumulation occurs and what it means for animal fitness during cancer cachexia.* 


*Reviewer #3 (Evidence, reproducibility and clarity (Required)):* 

*Summary:* 
*In this study, the authors use Drosophila larvae to investigate how epithelial tumors induce systemic metabolic changes characteristic of cancer cachexia. They focus on oenocytes-hepatocyte-like cells-and demonstrate that these cells accumulate lipid droplets in response to tumor-secreted factors Gbb and ImpL2. This lipid accumulation is modulated by metabolic alterations in distant tissues, including the fat body and muscle, revealing bidirectional non-autonomous crosstalk. The authors employ Gal4/UAS and QF/QUAS binary systems to independently manipulate tumors and target tissues. They show that changes in lipid synthesis, storage, or transport in the fat body or muscle influence oenocyte lipid content, and that oenocyte-specific manipulations can reciprocally affect fat body lipid stores. However, these oenocyte lipid changes do not influence muscle wasting, indicating that oenocytes are not central to muscle integrity during cachexia. The study also implicates reduced PI3K in oenocytes of tumor-bearing larvae as a regulator of oenocyte lipid levels. Overall, the work provides a nice view of inter-organ metabolic communication during tumor-induced wasting, showing how oenocytes are important modulators of systemic lipid status in tumor-bearing animals.* 

*Major comments:* 
*Overall this is a very well performed piece of work. The data are all well-presented and convincing, and the methods are clearly presented to allow other researchers to build on this work. Also, all the experiments adequately replicated and statistical analysis are excellent* 

*My only main comment concerns the result in Fig 5 showing increased FOXO in oenocytes of tumor bearing. The authors use this result to argue that these oenocytes have lower PI3/Akt activity. However, I think this result could be strengthened - FOXO is typically regulated by PI3k/AKT through control of its nuclear localization, i.e. mostly cytoplasmic when pI3k/Akt activity is high and strongly nuclear when PI3K/Akt activity is inhibited. In this context, the FOXO-GFP results in Fig 5C are a little tricky to interpret. The main effect seems to be a general overall increase in the FOXO-GFP levels in the tumor-bearing larvae rather than any change in foxo nuclear localization, in which case the result is difficult to interpret as a downregulation of PI3k/Akt.* 

*Can the authors clarify how they quantified this result? A higher magnification image without the added outlines of the nuclei would be helpful to interpret the result.*

*We have now presented the data as cytoplasmic: nuclear ratio and deleted the outlines of the nuclei.*

*Can they/did they look at cytoplasmic: nuclear staining of FOXO-GFP?* 
*We have presented the data as cytoplasmic:nuclear ratio, deleted the outlines of the nuclei and zoomed into the image.*

*The interpretation of this result would be strengthened with other additional measures of Pi3/Akt/FOXO signaling such as 1) direct staining of Foxo using a Foxo antibody (this isn't commercially available but is available from labs who work on Foxo)*

*or 2) staining for thor-LacZ - thor is a foxo target and so expression could be higher in the oenocytes from tumor animals,*

*or 3) the tGPH reporter (Britton et al 2003, Dev Cell).* 
*We have performed tGPH, in addition to trying Foxo antibody, which did not work well in our hands.*

*These additional experiments should take 2-3 months.* 

*Minor comments:* 

*- The introduction would benefit from a little more background on previous Drosophila studies looking at how disc tumors affect systemic lipid metabolism and fat/muscle wasting. This would help provide a little more context about the current work.*

*done*
*- Line 150. Should there be a sentence or explanation before, "To test this..." (to explain hat 'this' refers to*

*done*
*- Line 219 needs a reference to the relevant figure/panels.*

*This data has been withdrawn.*


*Reviewer #3 (Significance (Required)):* 

*Overall, the work makes an important conceptual addition to the field of tumor-host interactions. It builds upon previous work showing bi-directional communication between oenocytes and fat cells to show how these interactions operate in the context of a tumor model to control systemic lipid metabolism. Hence, it provides new insight into our knowledge of how tumor-host interactions modulate lipid biology.* 

*The primary factor that constrains the conceptual impact of the work is that manipulating oenocyte lipid metabolism didn't alter the overall cachectic phenotype. In this respect, the work, while excellent, doesn't provide as substantial a conceptual advance as other key papers in the field (including important and elegant work from the Cheng lab - e.g., Lodge et al Dev Cell 2021, Dark et al EMBO Rep 2024) that offer deeper insight into the signaling pathways and cellular mechanisms by which tumors affect both fat and muscle biology to induce tissue wasting. This limitation arises not from any shortcoming in the work or methodology but simply reflects that the biological processes uncovered do not directly connect to cachexia regulation.* 

*This work would be of interest to a number to fly researchers examining systemic physiology and metabolism and cancer researchers studying host-tumor interactions, as well as researchers generally interested in inter-organ communication and systemic lipid metabolism. Hence the work would be of broad interest.* 

*My area of expertise is in using Drosophila to study oncogene/tumor suppressor signaling pathways and their effects on growth and systemic physiology.*
