# Supplementary material for: Tumour-driven lipid accumulation in oenocytes reflects systemic lipid alterations
Source: PLoS Genet. 2026 May 7;22(5):e1012150. doi: 10.1371/journal.pgen.1012150 (PMC13167029; doi:10.1371/journal.pgen.1012150)
Supplement: Attachment — Submitted filename: rebuttal.docx [file pgen.1012150.s005.docx]

Dear Reviewer,

Thank you for your constructive suggestions. To address these concerns, we performed further experiments especially in regard to whether lipid accumulation is due to starvation. As the first author of the paper has left the lab, it is not possible for us to perform new exploratory experiments to address whether lipid accumulation plays a role in developmental contexts independent of cancer cachexia. While we agree this is an interesting topic, it is outside of the current scope of this paper. Finally, we have made editorial changes suggested, and have replaced images with more representative ones.

Yours sincerely,

Louise Cheng

1. From Figure 1, the authors show that lipid droplets begin to accumulate in oenocytes of tumor-bearing larvae starting around day 6 AEL. However, it is well established that a classical feature of oenocytes is the induction of lipid droplet accumulation in response to starvation. At approximately 6 days AEL during the larval stage, feeding activity is typically reduced or may even cease as larvae approach the wandering stage. Therefore, it would be important to consider whether the observed lipid droplet accumulation in oenocytes could at least partially result from reduced feeding or starvation, rather than being solely a consequence of tumor-induced cachexia. Clarifying the feeding status of the larvae at this stage, or including appropriate controls to distinguish starvation-induced lipid accumulation from tumor-associated effects, would strengthen the interpretation of these results.

To address this, we have performed new experiments, in which we compared lipid accumulation in WT, non-cachectic inducing tumour and cachectic-inducing tumour under Fed and Nutrient Restriction(NR) conditions (Figure 1). In animals carrying brain tumours caused by the knockdown of *prospero* (*pros*), which does not induce cachexia, but undergoes developmental delay, we found very little LD accumulation in the oenocytes (Figure 1K-M), suggesting that LD accumulation is a specific feature of cachexia-causing tumours. As LD accumulation has also been reported to occur during NR, we compared the level of LD accumulation in *Ras^V12^scrib^RNAi^* tumour bearing animals with wildtype (*w^1118^*) or *pros* tumour animals under NR. We found that *Ras^V12^scrib^RNAi^* tumour bearing animals tumour-bearing animals showed far higher LD accumulation under fed conditions than *w^1118^* or *pros* tumour animals under NR, suggesting while starvation is a possible contributor of lipid accumulation, the accumulation we observed in cachectic animals far exceeds that of starvation alone. Finally, we subjected *Ras^V12^scrib^RNAi^* tumour-bearing animals to NR and found this manipulation did not further increase lipid droplet accumulation, suggesting either that cachexia-inducing animals were already nutrient-starved even under fed conditions or alternatively, that cachexia induced lipid accumulation is dominant over NR-mediated lipid accumulation.

2. In addition, the authors introduced a neuroblast tumor model to address the concern raised by Reviewer 1. In their response, the authors state that Figures 1K–L do not show lipid droplet accumulation in oenocytes. However, this interpretation does not appear to fully match the images presented. When compared with the control shown in Figure 1C, the oenocytes in Figures 1K–L seem to contain a noticeably higher level of lipid droplets. Therefore, the current response does not convincingly demonstrate that the oenocyte lipid accumulation phenotype is specific to the cachectic tumor model.

We have repeated this experiment, this time we compared lipid accumulation in WT, non-cachectic inducing tumour and cachectic-inducing tumour under Fed and Nutrient Restriction(NR) conditions in parallel. In animals carrying brain tumours caused by the knockdown of *prospero* (*pros*), which does not induce cachexia, but undergoes developmental delay, we found very little LD accumulation in the oenocytes (Figure 1K-M), suggesting that LD accumulation is a specific feature of cachexia-causing tumours.

Furthermore, by Day 6 AEL, even control larvae (w1118) begin to display lipid droplet accumulation in oenocytes (Fig1.K). This observation raises the possibility that the phenotype may be influenced, at least in part, by reduced feeding or starvation at this stage rather than being exclusively tumor-dependent.

The fact that cachexia is preceded by anorexia is a well-established mechanism in cancer cachexia (Petsakou et al., 2026, Yeom et al., 2021). By comparing WT, non-cachectic and cachectic tumours under fed and NR, we found that the level of lipid accumulation observed in cachexia to far exceed that of NR in either WT or non-cachectic tumour bearing animals, suggesting that starvation while a likely contributor to this phenotype, cachexia is the main cause of lipid accumulation.

Starvation is also known to promote the secretion of ImpL2, which can induce systemic metabolic reprogramming under conditions of energy limitation. However, in the current study the authors do not directly demonstrate that ImpL2 is secreted from the tumors in their model, nor do they cite prior studies supporting this mechanism in the specific tumor context used here. Providing evidence for the source of ImpL2 would help strengthen the mechanistic interpretation of the study.

We beg to differ with the reviewer here, we believe the reviewer may have missed some of the data we have already presented. Tumour derived ImpL2 (as well as oenocyte derived ImpL2) are the focus of data we presented in Figure 1 and Figure S2. Furthermore, papers that describe the role of tumour derived ImpL2 (including some of our own studies) were extensively cited in our manuscript.

3. Although the authors have added scale bars and improved the statistical presentation in response to the reviewers’ suggestions, there still appears to be considerable room for improvement.
For instance, Figures 1B and 1K both purportedly show wild-type oenocytes, yet the cells exhibit a drastic difference in size. If the scale bars are indeed accurate, the authors must provide a biological or technical explanation for this substantial size discrepancy.

We have now corrected this mistake.

The sample sizes remain insufficient, particularly for biological processes as dynamic as severe lipid metabolic remodeling. Given the high degree of heterogeneity in fat body lipid droplet sizes, a larger sample population is essential to ensure the accuracy and reproducibility of the conclusions. For example, the LD area for wild-type controls is reported as approximately 90 um2 in Figure 2L, whereas it appears as only 25um2 in Figure 4K.

We have done more measurements for this particular experiment, we agree that the control LD size we presented was on the high side, additional samples have been replotted in 2L.

4. In Figures 2B–I, the authors knock down FASN1 and Bmm in the fat body. However, these two proteins play opposing roles in lipid storage: FASN1 is required for fatty acid synthesis, whereas Bmm functions as a lipase involved in triglyceride breakdown. Theoretically, the knockdown of Bmm should impair lipolysis and lead to an increase in lipid droplet size or triglyceride accumulation, as previously reported in several Drosophila studies. However, the authors report that knocking down either FASN1 or Bmm results in a similar phenotype. It would be helpful if the authors could clarify this point, or discussion to explain why suppression of a lipase produces a phenotype similar to that observed when lipid synthesis is inhibited.

We agree that Bmm inhibition in WT animals causes lipid droplet accumulation in the wildtype context. However, in the context of cancer cachexia, while it reduces the LD size, the changes in LD size in the fat body is not purely due to lipid accumulation. We have a paper under preparation, which shows that changes in lipid droplets in the fat body is actually reflective of changes in phospholipids, an integral component of the lipid droplet membrane. We have added some text in the discussion to address this point.

5. The analysis of the Gbb-induced TGF-β/BMP signaling pathway presented in this study appears to contribute only limited insight to the overall story. The connection between this pathway and the proposed model of systemic lipid redistribution or cachexia has not been sufficiently demonstrated. This section seems disconnected from the central narrative of the manuscript.

This was requested by a previous reviewer, since we have previously shown ImpL2 and Gbb are important tumour secreted ligands, it made sense to include this data. We have now moved the data where we examined the role of Gbb and ImpL2 from oenocytes into Figure S2.

**6.** The representative immunostaining images do not appear to closely match the corresponding quantitative results, which may cause confusion for readers.
For example, in Figure 2 (G and J), the lipid droplet staining in oenocytes visually appears much higher in panel G than in panel J. However, the quantified values shown in panel N are very similar for these two genotypes.
Similarly, in Figure 4 (D and H), the Actin staining appears visually quite different between the two panels, yet the corresponding quantification shown in panel M does not reflect any significant difference.

We have changed the representative images.

7. The authors should revise the schematic and the descriptions of hierarchical relationships in the manuscript. Based on the evidence currently provided, the data primarily demonstrate systemic metabolic reprogramming and changes in lipid distribution under cachexia. It is difficult to determine which of the three tissues, fat body, oenocytes, or muscle, occupies a central or upstream position in this network.
For example, the legend in Figure 2 describes oenocytes as being downstream of the fat body, which is not entirely accurate. In Figure 4, knockdown of FASN1 specifically in oenocytes also alters lipid distribution in the fat body. These results suggest a rearrangement of lipid distribution rather than upstream–downstream regulatory relationship between the tissues.

We agree with the reviewer that the upstream-downstream relationship can not be established through our analysis. We have now changed the title of the Figure legend 2 according to the reviewer’s suggestions. The final schematic summarising our findings already reflects the bi-directional crosstalk between fat body and oenocytes.

8. The reviewer raised an important question regarding whether this organ-to-organ communication is specific to the context of cachexia. I agree that this is a highly relevant and meaningful point. Systemic lipid redistribution is particularly important under conditions of nutritional stress, immune responses, or inflammation. If the authors could provide additional experiments to address whether similar mechanisms operate outside of the cachectic context, it would greatly enhance the significance and impact of the manuscript.

The first author of the paper has left the lab, while we agree this is an important point, we do not have the capacity to carry out further experiments to address what happens in the wildtype context, as investigating this phenomenon is beyond the scope of this manuscript, which focuses on the lipid mechanisms during cancer cachexia. This point was raised by reviewer 2, who was satisfied with the previous round of revisions.

**9.** Figure 2U-V genotype labelling in wrong order.

This has been fixed.
